# The effect of different dietary levels of DL-methionine and DL-hydroxy analogue on the antioxidant status of young turkeys infected with the haemorrhagic enteritis virus

**DOI:** 10.1186/s12917-018-1727-2

**Published:** 2018-12-17

**Authors:** Jan Jankowski, Bartłomiej Tykałowski, Katarzyna Ognik, Andrzej Koncicki, Magdalena Kubińska, Zenon Zduńczyk

**Affiliations:** 1 Department of Poultry Science, Faculty of Animal Bioengineering, University of Warmia and Mazury, Oczapowskiego 5, 10-719 Olsztyn, Poland; 2 Department of Poultry Diseases, Faculty of Veterinary Medicine, University of Warmia and Mazury, Oczapowskiego 13, 10-719 Olsztyn, Poland; 3 Department of Biochemistry and Toxicology, Faculty of Biology, Animal Sciences and Bioeconomy, University of Life Science in Lublin, Akademicka 13, 20-950 Lublin, Poland; 4 Division of Food Science, Institute of Animal Reproduction and Food Research of the Polish Academy of Sciences, Tuwima 10, 10-748 Olsztyn, Poland

**Keywords:** Haemorrhagic enteritis virus, Methionine, DL-methionine hydroxy analogue, Blood parameters, Antioxidant status, Turkeys

## Abstract

**Background:**

The results of experiments involving broiler chickens and turkeys indicate that increased dietary methionine (Met) levels may improve the antioxidant protection of tissues in fast-growing birds. This is an important consideration since viral infections induce oxidative stress. The aim of this study was to verify the hypothesis that turkey diets with increased Met content can suppress oxidation processes induced by infection caused by the haemorrhagic enteritis virus (HEV), and that the noted effect is determined by the chemical form of this amino acid: DL-methionine (DLM) or DL-hydroxy analogue of Met (MHA).

**Results:**

Dietary Met content above 40% higher than the level recommended by the NRC (1994) intensified lipid peroxidation in the small intestine, leading to an increase in malondialdehyde (MDA) and lipid peroxide (LOOH) levels, but it also stimulated antioxidant mechanisms in the blood and liver of turkeys infected with HEV. In comparison with DLM, MHA contributed to more severe symptoms of oxidative stress, such as elevated MDA levels in the intestines, and a decrease in glutathione peroxidase (GPx) activity and ferric-reducing ability of plasma (FRAP).

**Conclusions:**

In HEV-infected turkeys, diets with increased Met content did not exert a clear antioxidant effect, which was noted in uninfected birds. The prooxidant activity of Met observed in the small intestinal wall was suppressed in the blood and liver of turkeys, most likely due to intensified synthesis of uric acid and glutathione. In comparison with MHA, DLM had a more beneficial influence on the analysed parameters of the redox status in the small intestine, blood and liver of turkeys.

## Introduction

Due to the low level of methionine (Met) in natural feed ingredients, commercial poultry diets have been supplemented with feed-grade Met, mainly DL-methionine (DLM) or Met hydroxy analogue (MHA), as DL-2-hydroxy-4-(methyl) butanoic acid [30]. Experimental evidence has shown that many amino acids, including Met, play a dual role, nutritional and and immunostimulatory [1, 3, 4, 9, 10, 16, 17, 31]. According to a new concept, amino acids that participate in and regulate key metabolic pathways to improve health, survival, growth, development, lactation, and reproduction of organisms are classified as functional [32]. This group of amino acids includes sulphuric amino acids, mainly Met and cysteine [32], which have been found to alleviate the symptoms of intestinal oxidative stress [21]. The results of some studies [2, 5] indicate that significant health benefits can be obtained with very high dietary levels of Met, almost two-fold higher than those recommended to meet the growth needs of chickens. A beneficial influence of Met on the antioxidant status of turkeys was noted [11, 12, 24] when dietary Met content was around 50% higher than that recommended by the NRC [18].

Less severe symptoms of oxidative stress in the intestines in response to dietary Met supplementation, observed by Ruth and Field [21], may be an important consideration in viral and bacterial infections in poultry. Previous research has demonstrated that viral and bacterial infections lead to immunesuppression in poultry [14, 22] and induce oxidative stress in cells [13, 24, 27]. In an experiment performed on chickens infected with the Newcastle disease virus (NDV), multiple metabolic changes induced by oxidative stress were observed in tissues, including an increase in malondialdehyde (MDA) levels, and a decrease in reduced glutathione (GSH) concentrations and the activities of antioxidant enzymes (superoxide dismutase - SOD, glutathione peroxidase - GPx, glutathione reductase and glutathione S-transferase) [24]. In another study [13], laying hens infected with the Marek’s disease virus (MDV) were characterised by an increase in the levels of MDA and carbonyl derivatives (PC), and a decrease in GSH concentrations and total antioxidant status (TAS) in blood. Elevated MDA levels and decreased activities of antioxidant enzymes (SOD and GPx) were also noted in the blood of chickens infected with the avian infectious bronchitis virus (IBV) [27].

In view of the above, the results of recent studies seem interesting because they suggest that increasing dietary Met levels can improve immune and antioxidant protection in young clinically healthy turkeys [11, 12, 15, 34]. The above applies to diets supplemented with DLM and MHA, which chemically is not an amino acid but a metabolic precursor of DLM, readily converted to L-methionine when entering animal tissues [30]. Some experiments with chickens and turkeys [20, 26 29] have shown that MHA-supplemented diets, compared with DLM-dietary treatments, are distinguished by their effects on the improvement in antioxidant status of birds as manifested by increased total and reduced glutathione concentration in the liver. Therefore, the question arises whether increased addition of synthetic DLM or MHA to poultry diets could alleviate the symptoms of oxidative stress in birds exposed to viral infections.

The objective of this study was to verify the hypothesis postulating that turkey diets with increased Met content can suppress oxidation processes induced by infection caused by the haemorrhagic enteritis virus (HEV), and that the noted effect is determined by the chemical form of this amino acid: DL-methionine (DLM) or DL-hydroxy analogue of Met (MHA).

## Methods

### Birds and general management practices

The study was conducted on 120-d-old female Hybrid Converter turkey poults (Grelavi Co., Ketrzyn, Poland) with permission 45/2013 of the Local Ethical Committee for Animal Experiments located at University of Warmia and Mazury (Olsztyn, Poland). The birds were kept in separate isolated experimental boxes in the Pavilion of Avian Experimental Infections of safety class PCL 3, at the Department of Avian Diseases of the University of Warmia and Mazury in Olsztyn. A three-stage negative pressure cascade was maintained in the experimental boxes and passageways in the Pavilion. Air entering and leaving the building was passed through HEPA H13 filters to prevent uncontrolled cross-infections between groups and infections caused by environmental agents. All turkeys were vaccinated against turkey rhinotracheitis (TRT) at 1 day of age (Poulvac TRT, lot number: 21075D2, expiry date: 3 March 2017, administered by eye drop), against ND at 10 days of age (Nobilis ND Clone 30, lot number: A206BM01, expiry date: May 2017, administered by eye drop) and against Ornithobacterium rhinotracheale (ORT) at 28 and 49 days of age (Ornitin, lot number: 24811021A, expiry date: December 2016, administered by subcutaneous injection). Vaccination program was identical to that applied on most commercial turkey farms in Poland.

### Experimental design: Dietary treatments and experimental inoculation

The birds were randomly assigned to four dietary treatments, with 30 birds per group, treated with different levels and sources of supplemented Met. The total number of birds in one treatment was adapted to the size of pens, according to the density used in turkey rearing. For the physiological studies, 8 birds were selected from each treatment, commonly accepted as the minimum number of animals (turkeys) with a unified genotype that ensures reliable, reproducible and statistically significant results without repeating the procedure due to high intra-group variability.

Two sources of supplemental Met were used, DL-Met (Evonik Industries, Krefeld, Germany) or MHA (calcium salt of 2-hydroxy-4-(methyl) butanoic acid, Novus International, Inc., St. Lois, MO). The levels of dietary Met that were tailored to meet the NRC recommendations (1994) were regarded as “low” (L), and the treatments with increased Met dietary, by above 40% as proposed by some breeding companies was indicated as “high” (H). The nutritional value of basal diets (Table 1) was calculated according to the Polish Feedstuff Analysis Tables [25]. The analysed L or H Met content (including the equivalent amount of MHA) was 0.55 and 0.78% in weeks 1–4 of age, and 0.45 and 0.65% in weeks 5–8 of age, respectively. The preparation of experimental diets and the determination of the final dietary Met content are previously described by Jankowski et al. [11]. At 42 days of age, turkeys were experimentally inoculated with 1 ml of a suspension containing HEV at a dose of 10^4,3^ EID_50_, administered into the crop with a probe [14].

**Table 1:** Composition of basal diets (%) and calculated nutrient concentrations in basal diets

Item	Weeks 1–4	Weeks 5–8
Ingredients		
Wheat	28.61	33.67
Corn	20.00	15.00
Soybean meal	41.44	42.00
Rapeseed meal	–	2.50
Potato protein	4.00	–
Soybean oil	1.25	2.55
Sodium sulphate	0.15	0.15
Sodium chloride	0.17	0.15
Limestone	1.67	1.58
Monocalcium phosphate	2.00	1.59
L-Lysine HCl	0.34	0.38
L-Threonine	0.02	0.08
Choline chloride	0.10	0.1
Mineral-vitamin premix for turkeys^a^	0.25	0.25
Nutritional value^b^		
Metabolizable energy, MJ/kg^b^	11.89	12.36
Crude protein, %	27.0	25.0
Crude fibre, %	3.27	3.54
Ash, %	3.02	3.09
Lysine, %	1.74	1.60
Methionine, %	0.40	0.35
Methionine + cysteine, %	0.83	0.77
Threonine, %	1.05	0.98
Tryptophan, %	0.34	0.32
Calcium, %	1.20	1.10
Sodium, %	0.14	0.13

^a^0.5% of the premix provided per kg of diet: Vitamin A (all trans-retinol acetate) – 15,000 IU, vitamin D_3_ (cholecalciferol) – 5000 IU, vitamin E (all-rac-α-tocopheryl acetate) - 100 mg, vitamin K_3−_ 4 mg, vitamin B_1−_ 5 mg, vitamin B_2−_ 15 mg, vitamin B_6−_ 6 mg, niacin - 100 mg, biotin - 0.35 mg, pantothenic acid - 32 mg, nicotinic acid − 100 mg, folic acid - 4 mg, choline chloride - 700 mg, Mn - 100 mg, Zn - 80 mg, Fe - 60 mg, Cu - 20 mg, I - 1.5 mg, Se - 0.3 mg, Ca – 1.07 g

^b^Calculated according to the Polish Feedstuff Analysis Tables [25]

### Sample collection and analyses

Biochemical and antioxidant parameters were determined in blood, homogenates of the small intestinal wall and liver. Blood samples (5 ml) were collected into test tubes containing heparin (at a concentration of 20 IU/ml) and tissue samples were taken from 8 birds per group. Heparinised blood samples were centrifuged for 10 min × 3000 *g* at 4 °C, and plasma was stored at − 70 °C until analysis. An automatic biochemical HORIBA analyzer (Kyoto, Japan) was used for determination of plasma glucose (GLU), triacylglycerols (TAG), total cholesterol (TC), uric acid (UA), total protein (TP) and albumin (ALB) and the activities of alanine aminotransferase (ALT), aspartate aminotransferase (AST), alkaline phosphatase (ALP), creatine kinase (CK) and lactate dehydrogenase (LDH). As described previously [19], the following indicators of redox status were determined in the blood plasma, liver and small intestinal wall of turkeys: the concentrations of lipid peroxide (LOOH), malondialdehyde (MDA), the sum of reduced GSH and oxidised GSH (GSH + GSSG), the ferric-reducing ability of plasma (FRAP), vitamin C, superoxide dismutase (SOD) and catalase (CAT). The activities of SOD and GPx were determined in blood and tissues using Ransod and Ransel diagnostic kits (Randox Laboratories, Crumlin, UK).

### Statistical analysis

Statistical calculations were done with the aid of two-way ANOVA (Statistica 10.0 software) considering two main factors (dietary Met level effect and dietary Met source effect) as well as the interaction between those factors (level × source interaction). In tables, mean values (*n* = 8) with pooled SEM are shown, and the statistical significance was considered at *p* < 0.05.

## Results

### Redox parameters of the small intestinal wall

The applied dietary treatments affected selected parameters of the redox status in the small intestinal wall (Table 2). Turkeys fed diets with higher Met content were characterised by higher CAT activity (*P* = 0.008), lower SOD activity (*P* < 0.001), and higher levels of LOOH and MDA in the small intestinal wall (*P* = 0.002 and *P* < 0.001, respectively). Higher CAT activity (*P* = 0.009) and lower MDA concentrations (*P* = 0.043) were noted in the small intestinal wall of turkeys receiving DLM-supplemented diets compared with those fed MHA-supplemented diets. A Met dosage × source interaction (*P* = 0.004) was observed for SOD activity because the higher dietary DLM level decreased SOD activity, whereas the higher MDA level had no influence on SOD activity. A significant Met dosage × source interaction (*P* = 0.006) observed for MDA levels in the small intestinal wall of turkeys resulted from different effects exerted by dietary Met sources: DLM did not induce changes in MDA concentrations whereas the higher MHA level increased MDA concentrations.

**Table 2:** Redox parameters of the small intestinal wall in turkeys fed diets with different Met sources and content (*n* = 8)

	Redox parameters^5^				
	Vit C μmol/kg	CAT U/g protein	SOD U/g protein	LOOH μmol/kg	MDA μmol/kg
Treatment^1^					
DLM_L_	5.86	57.1	3.48^a^	5.34	1.04^b^
MHA_L_	5.44	50.6	2.36^b^	5.97	0.93^b^
DLM_H_	5.35	67.4	1.64^b^	6.70	1.08^b^
MHA_H_	5.86	57.3	2.15^b^	6.62	1.70^a^
Dosage^2^ (D)					
Low	5.65	53.9	2.92	5.66	0.99
High	5.61	62.4	1.90	6.66	1.39
Source^3^ (S)					
DLM	5.61	62.3	2.56	6.02	1.06
MHA	5.65	53.9	2.25	6.29	1.32
*p*-values					
D	0.872	0.008	0.001	< 0.001	0.002
S	0.872	0.009	0.249	0.260	0.043
D × S	0.116	0.556	0.004	0.149	0.006
SEM^4^	0.142	1.783	0.174	0.150	0.079

^1^Diets fed to turkeys containing DL-methionine (DLM_L_ and DLM_H_) and the equivalent amount of DL-methionine hydroxyl analogue (MHA_L_ and MHA_H_) at two levels: low (_L_) and high - (_H_)

^2^Low level - 0.55 and 0.45% and high level - 0.78, 0.65%, in weeks 1–4 and 5–8 week of feeding

^3^Sources of methionine: DL-isomer (DLM) and DL-hydroxy analogue of Met (MHA)

^4^SEM, standard error of the mean

^5^Vit C – vitamin C, CAT – catalase, SOD – superoxide dismutase, LOOH – lipid peroxides, MDA – malondialdehyde

^a-b^values differ significantly

### Biochemical and redox parameters of blood plasma

No significant differences were found in the analysed blood biochemical parameters of turkeys fed diets with different Met content and sources, except for plasma UA levels (Table 3), which increased with increasing inclusion levels of dietary Met (*P* = 0.019). A significant Met dosage × source interaction (*P* = 0.013) was also observed for plasma UA levels: the higher DLM level had no effect on the above parameter whereas the higher MHA level increased UA concentrations in the blood plasma of turkeys.

**Table 3:** Biochemical blood parameters in turkeys fed diets with different Met sources and content (*n* = 8)

	Biochemical blood parameters^5^										
	GLU mmol/l	TP g/l	ALB μmol/l	TAG mmol/l	TC mmol/l	UA μmol/l	ALT U/l	AST U/l	ALP U/l	CK U/l	LDH U/l
Treatment^1^											
DLM_L_	17.7	31.4	174	0.68	2.87	288^ab^	6.39	211	1412	2288	932
MHA_L_	18.0	28.7	190	0.50	2.62	226^b^	5.24	226	1447	2669	1164
DLM_H_	18.1	27.4	187	0.63	3.03	284^ab^	7.15	238	1325	2779	1101
MHA_H_	19.5	29.6	184	0.70	2.68	354^a^	5.49	228	1203	2261	1163
Dosage^2^ (D)											
Low	17.9	30.1	182	0.59	2.75	257	5.81	218	1430	2479	1048
High	18.8	28.5	186	0.66	2.85	319	6.32	233	1264	2520	1132
Source^3^ (S)											
DLM	17.9	29.4	181	0.66	2.95	286	6.77	225	1368	2534	1017
MHA	18.8	29.2	187	0.60	2.65	290	5.36	227	1325	2464	1163
*p-values*											
D	0.310	0.257	0.722	0.461	0.611	0.019	0.567	0.348	0.090	0.878	0.591
S	0.347	0.870	0.474	0.573	0.161	0.861	0.119	0.868	0.650	0.796	0.352
D × S	0.529	0.075	0.325	0.211	0.805	0.013	0.771	0.420	0.412	0.099	0.589
SEM^4^	0.431	0.699	4.563	0.049	0.103	14.46	0.437	7.653	47.85	131.0	75.60

^1^Diets fed to turkeys containing DL-methionine (DLM_L_ and DLM_H_) and the equivalent amount of DL-methionine hydroxyl analogue (MHA_L_ and MHA_H_) at two levels: low (_L_) and high - (_H_)

^2^Low level - 0.55 and 0.45% and high level - 0.78, 0.65%, in weeks 1–4 and 5–8 of feeding

^3^Sources of methionine: DL-isomer (DLM) and DL-methionine hydroxy analogue (MHA)

^4^SEM, standard error of the mean

^5^GLU: glucose, TP: total protein, ALB: albumin, TAG: triacylglycerols, TC: total cholesterol, UA: uric acid, AST: aspartate aminotransferase, ALT: alanine aminotransferase, ALP: alkaline phosphatase, CK: creatine kinase, LDH: lactate dehydrogenase

^a-b^values differ significantly

Multiple changes in the blood redox status were noted in response to different dietary Met levels and sources (Table 4). Dietary Met content had no influence on vitamin C concentrations, the activities of SOD, GPx and CAT or total antioxidant capacity determined in the FRAP assay. The higher Met level increased GSH + GSSG (*P* = 0.012) and LOOH (*P* = 0.035) concentrations, and tended to decrease MDA levels in the blood plasma of turkeys (*P* = 0.062). In comparison with DLM, MHA decreased vitamin C concentrations (*P* < 0.027) and GPx activity (*P* < 0.001), increased the activities of SOD and CAT (both *P* < 0.001), and decreased FRAP values and LOOH concentrations (*P* = 0.001). A significant Met dosage × source interaction was noted for some redox parameters: (1) the higher DLM level increased and the higher MHA level decreased GPx activity (*P* < 0.001), (2) the higher DLM level did not affect and the higher MHA level increased GSH + GSSG concentrations (*P* < 0.001), (3) the higher DLM level decreased and the higher MHA level increased FRAP values (*P* < 0.001).

**Table 4:** Blood redox parameters in turkeys fed diets with different Met sources and content (*n* = 8)

	Blood redox parameters^5^							
	Vit Cμmol/l	SOD U/g Hb	GPx U/g Hb	CAT U/g Hb	GSH + GSSG μmol/l	FRAP μmol/l	MDA μmol/l	LOOH μmol/l
Treatment^1^								
DLM_L_	5.66	306	147^b^	794	0.83^ab^	1108^a^	0.82	35.1
MHA_L_	4.95	385	143^b^	996	0.72^c^	588^c^	1.00	30.3
DLM_H_	5.32	265	173^a^	726	0.80^b^	854^b^	0.81	33.5
MHA_H_	4.90	377	107^c^	1119	0.89^a^	959^ab^	0.80	23.9
Dosage^2^ (D)								
Low	5.31	345	145	895	0.77	848	0.91	32.7
High	5.11	321	140	922	0.84	906	0.81	28.7
Source^3^ (S)								
DLM	5.49	286	160	760	0.81	981	0.82	34.3
MHA	4.92	380	125	1057	0.80	773	0.90	27.1
*p-value*								
Dosage	0.423	0.130	0.299	0.558	0.012	0.302	0.062	0.035
Source	0.027	< 0.001	< 0.001	< 0.001	0.634	0.001	0.139	< 0.001
D × S	0.559	0.303	< 0.001	0.066	0.001	*<* 0.001	0.088	0.190
SEM^4^	0.128	11.63	4.885	35.59	0.017	43.09	0.029	1.154

^1^Diets fed to turkeys containing DL-methionine (DLM_L_ and DLM_H_) and the equivalent amount of DL-methionine hydroxyl analogue (MHA_L_ and MHA_H_) at two levels: low (_L_) and high - (_H_)

^2^Low level - 0.55 and 0.45% and high level - 0.78, 0.65%, in weeks 1–4 and 5–8 of feeding

^3^Sources of methionine: DL-isomer (DLM) and DL- methionine hydroxy analogue (MHA)

^4^SEM, standard error of the mean

^5^Vit C: vitamin C, SOD: superoxide dismutase, GPx: glutathione peroxidase, CAT: catalase, GSH + GSSG: total glutathione, FRAP: ferric reducing ability of plasma, MDA: malondialdehyde, LOOH: lipid peroxides

^a-b^values differ significantly

### Redox parameters of the liver

Both experimental factors, i.e. the level and source of dietary Met, affected parameters of the redox status of the liver in turkeys (Table 5). Increased dietary Met content led to an increase in the activities of CAT and SOD (*P* = 0.009 and *P* < 0.001, respectively) and LOOH concentrations (*P* = 0.042), and a decrease in MDA levels (*P* = 0.002). In comparison with DLM, MHA decreased vitamin C concentrations and increased SOD activity (both *P* < 0.001) in the liver of turkeys. A significant Met dosage × source interaction (*P* = 0.039) was observed for CAT activity which did not change in response to the higher DLM level but increased in response to the higher MHA level. The higher MHA level had no influence on hepatic MDA concentrations, whereas the higher DLM level decreased MDA concentrations (Met dosage × source interaction, *P* = 0.026).

**Table 5:** Redox parameters of the liver in turkeys fed diets with different Met sources and content

	Redox parameters^5^				
	Vit C μmol/kg	CAT U/g protein	SOD U/g protein	LOOH, μmol/kg	MDA μmol/kg
Treatment^1^					
DLM_L_	158	334^b^	15.9	4.14	1.29^a^
MHA_L_	137	327^b^	17.4	3.89	1.11^a^
DLM_H_	146	346^b^	17.3	4.28	0.74^b^
MHA_H_	137	424^a^	17.9	4.73	1.01^ab^
Dosage^2^ (D)					
Low	148	330	16.7	4.01	1.20
High	141	385	17.6	4.50	0.88
Source^3^ (S)					
DLM	152	340	16.6	4.21	1.01
MHA	137	376	17.7	4.31	1.06
*p*-values					
D	0.125	0.009	< 0.001	0.042	0.002
S	< 0.001	0.079	< 0.001	0.662	0.598
D × S	0.127	0.039	0.087	0.141	0.026
SEM^4^	2.447	11.656	0.172	0.122	0.057

^1^Diets fed to turkeys containing DL-methionine (DLM_L_ and DLM_H_) and the equivalent amount of DL-methionine hydroxyl analogue (MHA_L_ and MHA_H_) at two levels: low (_L_) and high - (_H_)

^2^Low level - 0.55 and 0.45% and high level - 0.78, 0.65%, in weeks 1–4 and 5–8 of feeding

^3^Sources of methionine: DL-isomer (DLM) and DL- methionine hydroxy analogue (MHA)

^4^SEM, standard error of the mean

^5^Vit C: vitamin C, SOD: superoxide dismutase, CAT: catalase, LOOH: lipid peroxides, MDA: malondialdehyde

^a-b^values differ significantly

## Discussion

In previous studies, increased dietary Met levels improved the antioxidant protection of intestinal mucosa. Increased glutathione production, higher levels of total antioxidant capacity and reduced protein oxidation were observed in the intestinal mucosa of chickens fed DLM-supplemented diets [23]. Lower MDA levels were found in the duodenal mucosa of young turkeys fed MHA-supplemented diets [20].

In the present experiment, undesirable changes were noted in some redox parameters of the small intestinal wall in turkeys, including a decrease in SOD activity and an increase in LOOH and MDA concentrations and CAT activity, in response to diets with higher inclusion levels of dietary Met. Our results, which do not corroborate the findings of Park et al. [20] and Shen et al. [23], are consistent with previous research investigating the effects of Met and its derivatives on virus replication. It was found that L-Met is required for the replication of selected viruses, e.g. the Lansing strain of poliomyelitis virus and the PR8 strain of influenza A virus, whereas dietary DL-Ethionine, a structural analogue of DLM, inhibits the replication of the above viruses [8]. Methionine is a precursor of S-adenosyl methionine, the major methyl donor in the cell, responsible for DNA methylation [33]. DNA methylation protects viruses against the destructive effects of endonucleases by maintaining the integrity of genomic DNA, required for virus replication [8]. It was also found that the methyl group of Met was incorporated into the 5′-terminus of the mRNA of cytoplasmic polyhedrosis virus, thus promoting its replication [7]. Therefore, it appears that in our experiment, increased dietary Met content contributed to HEV replication in the intestines of turkeys, which intensified oxidative stress described in studies investigating the effects of viral and bacterial infections in poultry [13, 24, 27].

The results of earlier studies indicate that increased dietary Met levels exerted antioxidant effects in poultry, manifested by elevated plasma UA levels [6, 28] and enhanced activities of important antioxidant enzymes such as SOD and GPx in blood [26]. In our previous experiments performed on uninfected turkeys [11, 34], increased dietary Met levels improved the antioxidant parameters of blood plasma by increasing SOD activity, total glutathione levels and FRAP values. In the current study, a similar increase in dietary Met content increased plasma UA levels and improved some indicators of the blood redox status, i.e. increased total glutathione levels and decreased LOOH concentrations, but it did not increase the activities of antioxidant enzymes or FRAP values.

In the present experiment, an increase in UA levels and considerable changes in the analysed redox status parameters were noted in the blood plasma of turkeys, possibly due to the antioxidant activity of Met (higher in the case of DLM) and oxidative stress caused by HEV infection. The presence of oxidative stress in cells and tissues, induced by bacterial and viral infections in poultry, has also been reported by other authors. The symptoms of oxidative stress included an increase in MDA levels, and a decrease in GSH concentrations and the activities of antioxidant enzymes [24], an increase in the levels of MDA and carbonyl derivatives (PC), and a decrease in GSH concentrations and TAS in blood [13], a decrease in the activities of antioxidant enzymes, and an increase in MDA levels [32].

In another experiment with uninfected turkeys [34] fed identical diets to those administered in the present study, increased dietary Met content contributed to an increase in SOD activity, glutathione concentrations and FRAP values. Based on literature data [13, 24, 27], it can be assumed that oxidative stress caused by HEV infection reduced the antioxidant effects of higher dietary Met levels in our study. However, a significant impairment in the redox status was observed in the small intestine, but not in the blood or liver of turkeys. This probably resulted from elevated plasma levels of UA and GSH, because UA, the product of the catabolism of proteins with unbalanced amino acid composition [6], is a highly effective antioxidant in the blood of poultry [28]. Elevated plasma UA levels, observed in our study, point to increased UA synthesis in the liver, most likely due to dietary amino acid imbalance [6]. Methionine is also a precursor for the synthesis of cysteine, an amino acid required for glutathione synthesis [3]. Some experiments with chickens and turkeys [20, 26, 29] have shown that MHA-supplemented diets, compared with DLM, have a better antioxidant status reflected in a lower rate of lipid peroxidation, probably due to higher hepatic concentrations of total and reduced glutathione. In the current study, MHA did not exert a more beneficial influence on turkeys than DLM. Turkeys fed MHA-supplemented diets were characterised by lower CAT activity and higher MDA levels in the small intestinal wall, which increased with increasing MHA doses. Impairment in the redox status was also noted in blood, although to a lower extent because a decrease in FRAP values was not accompanied by an increase in LOOH or MDA levels. The least pronounced differences in the prooxidant and antioxidant effects of DLM and MHA were found in the liver of turkeys.

The results of this study indicate that dietary Met content approximately 40% higher than the level recommended by the NRC (1994) intensified lipid peroxidation in the small intestine, leading to an increase in MDA and LOOH levels, but it also stimulated antioxidant mechanisms in the blood and liver of turkeys infected with HEV. In comparison with DLM, MHA increased the activities of SOD and CAT, and decreased GPx activity and FRAP values.

## Conclusions

In HEV-infected turkeys, diets with increased Met content did not exert a clear antioxidant effect, which was noted in uninfected birds. The prooxidant activity of Met observed in the small intestinal wall was suppressed in the blood and liver of turkeys, most likely due to intensified synthesis of uric acid and glutathione. In comparison with MHA, DLM had a more beneficial influence on the analysed parameters of the redox status in the small intestine, blood and liver of turkeys.

## Abbreviations

ALB: albumin
ALP: alkaline phosphatase
ALT: alanine aminotransferase
AST: aspartate aminotransferase
CAT: catalase
CK: creatine kinase
DLM: DL-methionine
FRAP: ferric reducing ability of plasma
GLU: glucose
GPx: glutathione peroxidase
GSH + GSSG: total glutathione
HEV: haemorrhagic enteritis virus
LDH: lactate dehydrogenase
LOOH: lipid peroxides
MDA: malondialdehyde
Met: methionine
MHA: DL-methionine hydroxy analogue
SOD: superoxide dismutase
TAG: triacylglycerols
TC: total cholesterol
TP: total protein
UA: uric acid
Vit C: vitamin C
